# Greater New York’s Triumph

**Published:** 1899-10

**Authors:** 


### EDITORIAL.

### GREATER NEW YORK'S TRIUMPH.

THE meeting in New York has come and gone, and what a wealth of delightful memories it has left in its train! Who will dare to stand up and say for a moment that he prophesied such an outpouring of the profession as was noted on this splendid occasion? More veterinarians in attendance than had ever gathered together at one time and place in the history of the profession of North America, and perhaps of the whole world.

The dawning day of the autotrucks and automobiles found in the Metropolitan City in September the greatest number, the most sanguine body, the most hopeful aggregation of professional men, whose interests are so closely allied with the horse industry, that made the thought of a horseless age the veriest dream of those dreamers who are ever soaring in chimerical areas and filling the world with air castles of pleasure and delight, and who die ignorant and strangers to the fleeting care-destroyers and comforts of the equine age. They were there to tell of the better days in their profession; of the advancing values in the equine market; of the new sphere of the horse and his future greater need as man's reliance and companion, and there to study the questions of how best to preserve his usefulness; conserve the wants of those who seek even higher classed specimens of the equine family, and no thought of a future day when this noble animal will be ignored, but oblivious of any danger from the stock-jobbing schemes of promotors of value on paper—to shear the unwary.

Successful beyond the thought of any in numbers, it was also a meeting that brought forth a programme more than rich with excellent papers, reports, and timely suggestions of matters very pertinent to the rapid advances along sanitary lines and the newer fields of bacteriological research and the laboratories' contributions to clearer scientific views and methods.

The old story must again be repeated of New York as it has been of other convention cities, that too much time was given to the local committee on the entertainment side; and while New York outdid herself in furnishing pleasure and enjoyment to all, it was again at the expense of ignoring wholly many valuable papers, curtailing others, and allotting but a few minutes for the discussion of subjects of the greatest national and State importance that filled many with concern, and was a source of regret and disappointment to a large number who had travelled hundreds of miles to hear and consider these topics. This places before the Executive Committee one of two perplexing problems: either that of an extension to four days' sessions or a curtailment of the time allotted for entertainment by the local veterinarians. Which will it be?

